# A missense variant in *CST3* exerts a recessive effect on susceptibility to age-related macular degeneration resembling its association with Alzheimer’s disease

**DOI:** 10.1007/s00439-015-1552-7

**Published:** 2015-04-19

**Authors:** Joe M. Butler, Umar Sharif, Manir Ali, Martin McKibbin, Joseph P. Thompson, Richard Gale, Yit C. Yang, Chris Inglehearn, Luminita Paraoan

**Affiliations:** Department of Eye and Vision Science, Institute of Ageing and Chronic Disease, University of Liverpool, Liverpool, L69 3GA UK; Ophthalmology and Neuroscience, University of Leeds, Leeds, LS9 7TF UK; Ophthalmology Department, St James’s University Hospital, Leeds, LS9 7TF UK; Ophthalmology Department, The York Hospital, York, YO31 8HE UK; Ophthalmology, The Royal Wolverhampton NHS Trust, Wolverhampton, WV10 0QP UK

## Abstract

Age-related macular degeneration (AMD) and Alzheimer’s disease (AD) are degenerative, multifactorial diseases involving age-related accumulation of extracellular deposits linked to dysregulation of protein homeostasis. Here, we strengthen the evidence that an nsSNP (p.Ala25Thr) in the cysteine proteinase inhibitor cystatin C gene *CST3*, previously confirmed by meta-analysis to be associated with AD, is associated with exudative AMD. To our knowledge, this is the first report highlighting a genetic variant that increases the risk of developing both AD and AMD. Furthermore, we demonstrate that the risk associated with the mutant allele follows a recessive model for both diseases. We perform an AMD-*CST3* case–control study genotyping 350 exudative AMD Caucasian individuals. Bringing together our data with the previously reported AMD-*CST3* association study, the evidence of a recessive effect on AMD risk is strengthened (OR = 1.89, *P* = 0.005). This effect closely resembles the AD-*CST3* recessive effect (OR = 1.73, *P* = 0.005) previously established by meta-analysis. This resemblance is substantiated by the high correlation between *CST3* genotype and effect size across the two diseases (*R*^2^ = 0.978). A recessive effect is in line with the known function of cystatin C, a potent enzyme inhibitor. Its potency means that, in heterozygous individuals, a single functional allele is sufficient to maintain its inhibitory function; only homozygous individuals will lack this form of proteolytic regulation. Our findings support the hypothesis that recessively acting variants account for some of the missing heritability of multifactorial diseases. Replacement therapy represents a translational opportunity for individuals homozygous for the mutant allele.

**Electronic supplementary material:**

The online version of this article (doi:10.1007/s00439-015-1552-7) contains supplementary material, which is available to authorized users.

## Introduction

Age-related macular degeneration (AMD) and Alzheimer’s disease (AD) are progressive neurodegenerative diseases exhibiting some common characteristics. A physical characteristic of both diseases is the presence of insoluble deposits at the site of pathogenesis. These pathological deposits—the amyloid plaques of AD and the drusen of AMD—demonstrate some compositional similarity, engender a pro-inflammatory response and impair essential cellular functions such as trafficking and secretion. These similarities indicate that common/similar cellular mechanisms may contribute towards the pathogenesis of both diseases. Certain environmental risk factors, such as smoking and obesity, are known to increase the risk of both diseases, along with age which is the major risk factor for both conditions. With respect to genetic risk factors the *APOE* gene is associated with both diseases but quite intriguingly has opposing directions of effect. Whereas the APOE *ε4* allele increases risk of developing AD, it decreases the risk of AMD (Baird et al. 2006; Logue et al. 2014; McKay et al. 2011).

A polymorphism in the cystatin C gene (*CST3*) has also been implicated as a risk factor for both AD (Hua et al. 2012) and AMD (Zurdel et al. 2002). The *CST3* polymorphism associated with both diseases is a non-synonymous SNP (rs1064039) in the signal sequence (p.Ala25Thr due to a c.G73A substitution) which results in an alternate homologue referred to as variant B. Cystatin C is a potent inhibitor of cysteine proteases and multiple lines of evidence (from molecular studies) support the hypothesis that wild-type cystatin C has a protective role against both these age-related diseases (Kaeser et al. 2007; Mi et al. 2007).

Meta-analysis of 8 association studies has confirmed that this SNP is associated with AD in Caucasians (Hua et al. 2012). Individuals homozygous for the variant were found to be at greatest risk (OR_AA_ = 1.73, *P* = 0.005), while heterozygous individuals were not at significantly increased risk (OR_AG_ = 1.06, *P* = 0.50), indicating that the risk allele acts recessively (Fig. S1). The genetic association between *CST3* and AMD has been less well studied, with only a single case–control study reported to date, in which an association between exudative AMD and the polymorphism was highlighted (Zurdel et al. 2002). Mirroring the AD association, Zurdel’s study found that it was those individuals homozygous with the variant that were found to be at the greatest risk of exudative AMD (OR_AA_ = 3.03, *P* = 0.01), whereas heterozygotes were not at significant risk (OR_AG_ = 1.06, *P* = 0.76). This identical recessive effect of *CST3* on AMD and AD risk is intriguing. In the line of the above, the main aim of this study was to further investigate the AMD-*CST3* association.

## Methods

### Association study subjects and ethics

A total of 350 Caucasian exudative AMD patients (126 males and 224 females) were recruited (age range 65–96 with mean 80.1 years). Written informed consent for all participants used in this study was obtained for research use and approved by the Leeds (East) Research Ethics Committee. The diagnosis of exudative AMD was provided by ophthalmologists based on baseline stereoscopic colour fundus, fluorescein and indocyanine green angiogram images to identify lesion characteristics (McKibbin et al. 2012). Inclusion criteria for the study were that the patients were aged 65 years and over, with choroidal neovascularization (CNV) secondary to AMD and involving the centre of the fovea, and with the CNV occupying more than 50 % of total lesion area. Patients that had CNV secondary to pathological myopia, inflammatory disease, angioid streaks or trauma were excluded from this study. Tests for dementia were not performed on these cases.

Population controls were taken from the largest publicly available online database Exome Variant Server, NHLBI GO Exome Sequencing Project (ESP), Seattle, WA (http://evs.gs.washington.edu/EVS/) (January 2014). This provided genotype information for 3781 Caucasians from the USA, which are assumed to contain undiagnosed AMD cases with a frequency equivalent to the Caucasian prevalence. We inferred that 2442 (64.5 %) of this sample are male, in that they have genotype information for the SRY gene.

### Genotyping

Genomic DNA was extracted from peripheral blood leucocytes by standard methods. Primers were designed using the online software Primer3 v.0.4.0 (http://frodo.wi.mit.edu/). Polymerase chain reaction (PCR) generated a 1292-bp product using forward primer CST3LRIIF 5′-CAGGAGTGGAGGAGGGAGATG-3′ and reverse primer CST3LRIIR 5′-CCAGATGAGGGGCTCTGTTTT-3′. This product contains three SNPs (rs5030707, rs73318135 and rs1064039) in strong linkage disequilibrium, such that the genetic variation can be explained by two haplotypes, known as variant A and variant B. Two of the SNPs are located in the 5′ untranslated region and the third is located in exon 1 (leading to the missense p.A25T). Briefly, the PCR consisted of 40 ng of genomic DNA, 2pM of each forward and reverse primer, 1M Betaine and HotShot Mastermix (Clent Life Sciences, Stourbridge, UK). An initial denaturation step of 95 °C for 12 min was followed by 40 cycles of 94 °C for 30 s, 60 °C for 30 s and 72 °C for 60 s. A final extension of 75 °C for 5 min completed the reaction. PCR products were electrophoresed on a 1.5 % agarose gel stained with ethidium bromide after which the gel was visualized using the ultraviolet light filter on the ChemiDoc Imaging system (BioRad).

### Sanger sequencing

PCR products were digested with ExoSAP-IT (Affymetrix USB, Santa Carla, USA) and sequencing reactions were carried out using Big Dye Terminator Cycle Sequencing V3.1 Ready Reaction Kit (Applied Biosystems, Warrington, UK). To determine the sequence at SNPs rs5030707, rs73318135 and rs1064039 nested reverse CST3LRR primer 5′-GGCTCCTGGAAGCTGATCTTAG-3′ was used. To confirm the sequence a second nested reverse primer CST3BIIR 5′-TTGCTGGCTTTGTTGTACTCGC-3′ was used. The sequence data obtained from both primers was compared to see if they matched and together these data were used to determine the haplotypes. The sequencing reactions were run on an ABI3130xl Genetic Analyser and the data analysed for respective SNPs using Sequence Analysis 5.2 software (Applied Biosystems). Representative chromatograms of each of the three genotypes are presented in Supplementary Fig. S2.

### Statistical analyses

The odds ratios and 95 % confidence intervals are log transformed to determine the mean and variance corresponding to the asymptotically normally distributed effect sizes (denoted as *β*). By calculating these parameters for both the heterozygote (*β*_AG_) and homozygote (*β*_AA_), we are able to make inferences about the genetic model of inheritance. Explicitly we test for the recessive model by testing the null hypothesis *H*_0_: *d* = *β*_AA_ − *β*_AG_ = 0, *H*_a_: *d* > 0 as previously described (Bagos 2008).

To summarize the level of homogeneity between AMD and AD effect sizes across both genotypes, we calculate the coefficient of determination from the four estimated ORs. We also test the null hypothesis that *CST3* has no effect on both diseases, or equivalently that the mean effect size is zero ($H_{0} :\beta_{\text{AMD}} = \beta_{\text{AD}} = \overline{\beta } = \, 0$). Here, the weighted mean and variance of the mean (using inverse-variance weighting) are used to determine the appropriate *z*-score and corresponding *P* value.

To test whether there is a significant difference in the distribution of three genotypes between AMD cases and controls we performed a two-sided Fisher’s exact test, conducted in R (R Core Team 2014). Meta-analysis was performed using Cochrane Review Manager with Mantel–Haenszel estimation (The Cochrane Collaboration 2012). Random effects meta-regression was performed in R using the ‘glmer’ function from the lme4 package.

### Power calculations for AMD association studies of CST3 variant

To determine the power of the association study of Zurdel et al. (2002) a single iteration randomly allocates a genotype (“AA”, “AG” or “GG”) to 517 simulated controls and 167 simulated cases, the sample sizes of this study. The probabilities used to allocate are calculated from the alternative hypothesis effect sizes, which is taken to be that reported by the AD meta-analysis (OR_AG_ = 1.06, OR_AA_ = 1.73). From this simulated case–control dataset we perform a two-tailed *z* test (on the logOR_AA_ scale) at *α* = 0.05. After 10,000 iterations the number that successfully detected an association is used to estimate power. This is repeated for our study by changing the sample sizes of cases and controls accordingly. For the two-study meta-analysis, power calculation requires simulating the two case–control data sets for each iteration and performing the *z* test based on the weighted normal distribution (equivalent to a fixed-effect meta-analysis).

## Results

### Recessive effect of *CST3* variant previously observed in both AD and AMD

**Fig. 1:**
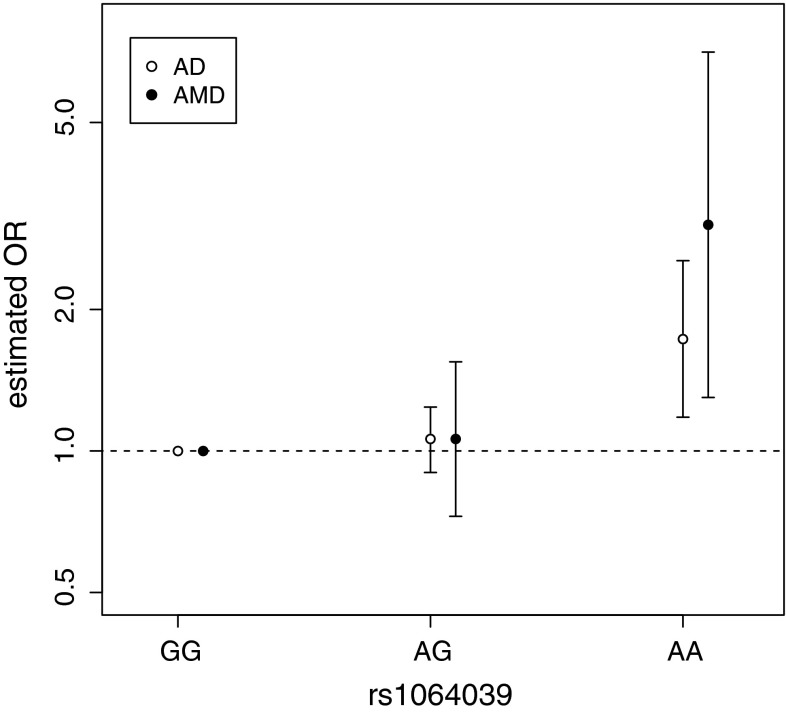
Odds ratios for *CST3* genotypes at rs1064039 estimated for AD by meta-analysis and for AMD by a single association study. ORs are measured relative to the “GG” genotype, by definition this baseline genotype has an OR of 1. *Error bars* represent 95 % CIs

Association between the *CST3* SNP (rs1064039) and AD has been established by meta-analysis (Hua et al. 2012). The exact same SNP has also been identified to be associated with AMD (Zurdel et al. 2002). Using the data from both these studies, we calculate the effect sizes separately for heterozygotes “AG” and homozygotes “AA”, against the baseline “GG” (Fig. 1, Fig. S1). We observe that for both diseases, risk is significantly increased only for the homozygotes (AD: OR_AA_ = 1.73, *P* = 0.005; AMD: OR_AA_ = 3.03, *P* = 0.01), whereas the heterozygote risk is non-significant for both diseases (AD: OR_AG_ = 1.06, *P* = 0.50; AMD: OR_AG_ = 1.06, *P* = 0.76). Thus a recessive model of inheritance best explains the association with *CST3* for both diseases. To support this recessive model we confirm that homozygote effect size is significantly greater than the heterozygote effect size in both AD (*P* = 0.010) and in AMD (*P* = 0.013). Put together the risk “A” allele is recessive for both diseases; only individuals with two copies of it are at a significantly higher risk of developing AD and AMD.

To quantify the similarity between the OR_AA_ for AD and AMD, we also calculated how probable it would be to simultaneously observe both these ORs by chance given the null hypothesis that *CST3* has no effect on both diseases. We find that such a set of observations is very unlikely to happen by chance (*P* = 5.0 × 10^−4^). To further quantify the similarity between the *CST3* genotype data of the two diseases, we calculate the coefficient of determination of the four variables (Fig. 1) and find *R*^2^ = 0.673.

### Power of existing association studies to detect *CST3* recessive effect is estimated to be low

To estimate the power of an association study an estimate of the effect size of the alternative hypothesis is required. Because both molecular and epidemiological evidence support homogeneity between AMD and AD with respect to *CST3*, we use its AD effect size estimated by meta-analysis (OR_AA_ = 1.73) as a reasonable estimate for its AMD effect size. Using this assumption and a *z* test for recessive effect we calculate the power of Zurdel’s study (167 cases, 517 controls) to be 24.6 %. Thus for every four studies of such size, only one would detect the association.

**Fig. 2:**
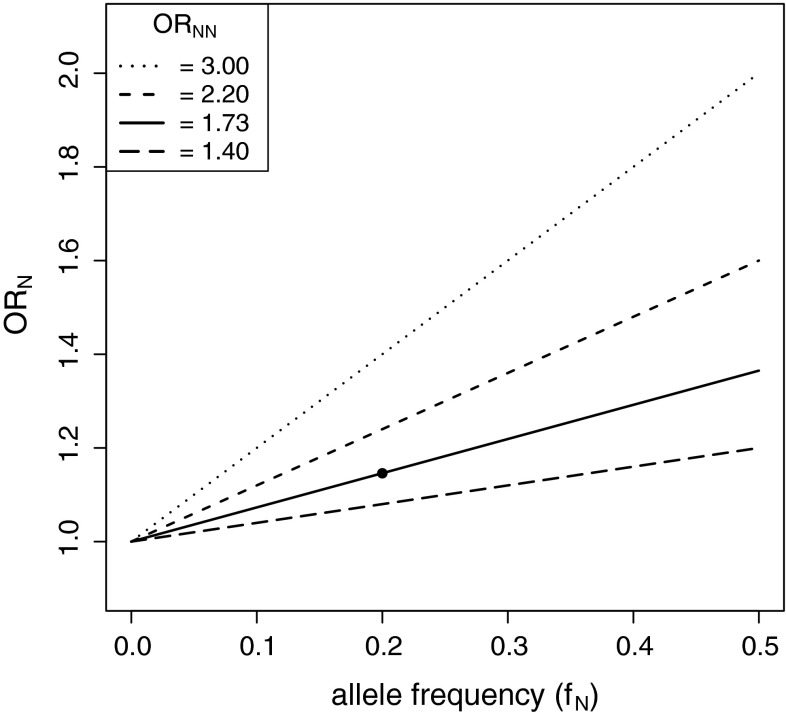
The per-allele odds ratio (OR_N_) decreases linearly with decreasing allele frequency (*f*_N_) when the true model is recessive; the elevated risk of homozygotes is kept constant (OR_NN_ specified) and heterozygotes are at baseline risk (OR_NX_ = 1). This relationship can be expressed as: OR_N_ = *f*_N_(OR_NN_ − 1) + 1. The *single point* represents *CST3* rs1064039 with respect to AD

We also estimated the power of a GWAS to detect an association with a variant with this recessive effect size. Using the sample sizes, test and significance level of an existing AD GWAS (Harold et al. 2009), we estimated the power to be 14.8 %. One reason for this low power is that the standard GWAS uses a test based on an additive model. This test performs poorly when the true causal variant is recessive (Lettre et al. 2007). We calculated that the per-allele (or additive model) odds ratio, OR_A_, would be 1.15 given a true recessive effect of OR_AA_ = 1.73 (given the allele frequency of rs1064039 and HWE in controls). From further study of this relationship we found that for a given recessive effect size, OR_NN_, the perceived per-allele OR_N_ is linearly related to the allele frequency of the SNP (Fig. 2). Thus even a variant with a large recessive effect (OR_NN_ = 3) and moderate allele frequency (10 %) can appear to have a weak effect from its per-allele OR (OR_N_ = 1.2).

### Novel AMD-*CST3* case–control study consistent with recessive effect

On observing these findings we sought to replicate the finding of Zurdel et al. in investigating the association between *CST3* and AMD. The *CST3* SNP (rs1064039) was genotyped in Caucasian AMD patients from England (*n* = 350). We tested this AMD data against the Exome Sequencing Project control data as it was the largest publically available set of population controls (*n* = 3781). In this control sample the frequency of the variant allele “A” is 17.5 % and the proportion with the “AA” genotype is 3.0 %. Thus the data are in Hardy–Weinberg equilibrium (*P* = 0.76) and also fall within the allele frequency range reported from the Caucasian studies in the AD meta-analysis, which ranged from 17.1 to 22.8 %.

Case–control analysis of these results exhibits a highly similar pattern of genotype risks to those observed by Zurdel (Table 1), but is not significant at an alpha level of 0.05 (two-sided Fisher’s exact test: *P* = 0.25). Although not significant, it is the “AA” homozygotes that are at greatest risk (OR_AA_ = 1.56, *P* = 0.11) compared to the heterozygotes (OR_AG_ = 1.07, *P* = 0.58), with “GG” homozygotes as baseline. Thus our data are consistent with the recessive effect observed previously in both AMD and AD, but are not powerful enough to reach significance by itself. Further indication of an effect was obtained by performing the analysis only on AMD cases aged above 80 years (OR_AA_ = 2.05, *P* = 0.03, *n* = 188). However, all further analyses in this study are performed on the total AMD dataset (i.e. ≥65 years, *n* = 350).

**Table 1:** Distribution of *CST3* rs1064039 genotypes in exudative AMD case and control samples from Caucasian population

Genotypes	Frequencies (%)		OR^a^ (95 % CI)	*P* value
	Case	Control		
G/G (baseline)	230 (65.7)	2574 (68.1)	1	–
G/A	104 (29.7)	1092 (28.9)	1.07 (0.84–1.36)	0.58
A/A	16 (4.6)	115 (3.0)	1.56 (0.91–2.67)	0.11

^a^Odds ratio were calculated separately against G/G baseline genotype

We calculate the power of our study alone to be 53.7 %, meaning that around half of studies this size would fail to detect the association given the effect size reported for AD. The relatively low powers presented so far are likely due to the frequency of the homozygote risk genotype. For instance within our sample of 350 AMD cases, only 16 (4.6 %) are “AA” homozygotes (Table 1). To achieve a power of 80 % (assuming the effect size is equivalent to AD, OR_AA_ = 1.73), we calculate it would require a sample of 735 AMD cases, whilst maintaining the control sample size of 3781.

### Combining AMD-*CST3* studies strengthens evidence of a recessive effect

**Fig. 3:**
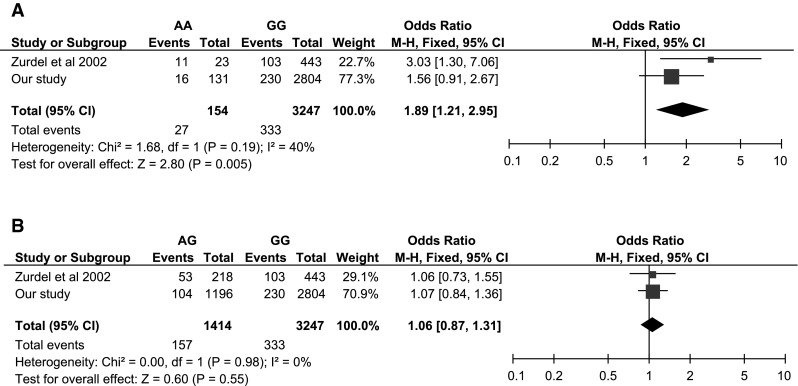
Forest plots for the meta-analysis of *CST3* rs1064039 with respect to exudative AMD in the Caucasian population using a fixed effects model. Size of the *squares* represents the weight of the study and *horizontal bars* represent 95 % CI of the OR. Applied to **a** “AA” genotype versus “GG” genotype and **b** “AG” genotype versus “GG” genotype

We proceeded to perform a preliminary meta-analysis to bring together the results of the two *CST3*-AMD association studies. First we apply a fixed-effects meta-analysis to the “AA” genotypes versus the baseline “GG” and determine a significant effect (OR_AA_ = 1.89, *P* = 0.005) (Fig. 3a). We estimate the power of this two-study meta-analysis to be 67.7 %, greater than either of its constituent association studies as expected. Thus, taken together, the two association studies indicate a significant overall recessive effect of *CST3* genotype on AMD risk. We also performed the meta-analysis using a random effects analysis and with this more conservative method the significant recessive effect is maintained (OR_AA_ = 2.00, *P* = 0.032). We also repeated the random effects meta-analysis using a meta-regression approach (Turner et al. 2000), and found the results matched well (OR_AA_ = 2.17, *P* = 0.026) with the conventional random effects meta-analysis.

**Fig. 4:**
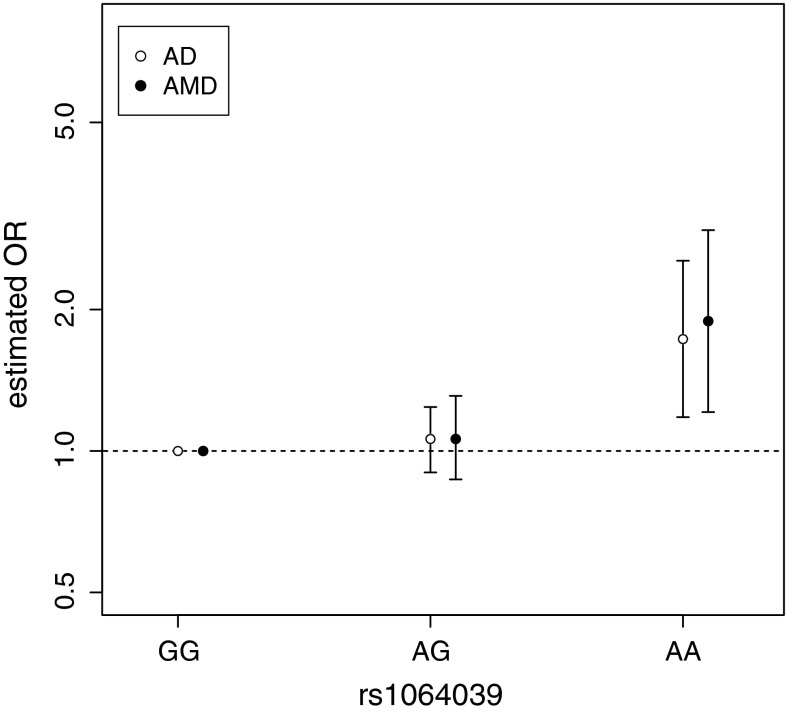
Odds ratios for *CST3* genotypes at rs1064039 estimated for AD and AMD meta-analyses. Note that the odds ratios are measured relative to the “GG” genotype, by definition this baseline genotype has an odds ratio of 1. *Error bars* represent 95 % CIs

We calculated how probable it would be to simultaneously observe both OR_AA_ under the null hypothesis of *CST3* having no effect on either disease. We found that this updated set of observations was even more unlikely to happen by chance (*P* = 7.8 × 10^−5^) than previously calculated. We then applied an AMD meta-analysis to the “AG” heterozygotes versus the baseline “GG” genotype (Fig. 3b), and determined a non-significant effect (OR_AT_ = 1.06, *P* = 0.55). Finally, we compared the AMD and AD effect sizes estimated from their respective meta-analysis alongside one another and observed a striking similarity (Fig. 4). Using the updated AMD effect sizes we found that the coefficient of determination now becomes very high (*R*^2^ = 0.978), supporting the hypothesis that homogeneity exists between AMD and AD risk with respect to *CST3* genotype.

## Discussion

We bring together AD and AMD case–control data and observe that not only is *CST3* associated with both diseases but there is a striking similarity in the underlying model of inheritance, namely a recessive genetic model. We first noticed this similarity by bringing together an AD-*CST3* meta-analysis and the only reported association study between *CST3* and AMD. Under the null hypothesis that both diseases are not affected by *CST3* genotype the combined observed data are very unlikely to occur by chance (*P* = 5.0 × 10^−4^). However, we estimated the power of this AMD association analysis to be fairly low (24.6 %), assuming the AMD effect size is equivalent to AD. On repeating the AMD association study, again the same recessive trend was observed with only the homozygote variants at elevated risk. Taken together a meta-analysis of the two AMD-*CST3* studies finds a significant association (*P* = 0.005) with an increased estimated power of 67.7 %. The recessive trend is strikingly similar between the two diseases (Fig. 4), with only the “AA” homozygotes at a significantly elevated risk of developing both AMD and AD, whereas the heterozygotes are non-significant and effectively equivalent in both diseases. The combined dataset of all AMD and AD studies is now even more unlikely to occur by chance (*P* = 7.8 × 10^−5^) given the null hypothesis that *CST3* has no effect on both diseases.

Although an estimated power of 67.7 % was achieved through the two-study meta-analysis, more replication association studies are necessary to validate a role of *CST3* in AMD pathogenesis. It is also important to note that both of these AMD association studies were performed with Caucasian samples only. With AD the association with *CST3* was only found in Caucasian samples, while in Asian samples no significant AD-*CST3* association was detected (Hua et al. 2012). Whether this ethnic disparity also translates across to AMD remains to be determined. A further aspect of the AMD-*CST3* association that remains to be unravelled is whether there is any epistasis between *CST3* and other known AMD genetic risk factors such as *CFH*, *ARMS2* and *APOE*.

We are aware that GWASs of AMD have failed to report an association at *CST3* (Arakawa et al. 2011; Chen et al. 2010; Cipriani et al. 2012; Fritsche et al. 2013; Neale et al. 2010; Yu et al. 2011). However, the fact that it has not reached genome-wide significance does not preclude it as a risk variant. This is demonstrated by the fact that the AD-*CST3* association, validated by candidate gene meta-analysis (Hua et al. 2012), has also not been reported in any GWAS for AD (Harold et al. 2009; Hollingworth et al. 2011; Lambert et al. 2009; Naj et al. 2011; Seshadri et al. 2010), nor a GWAS meta-analysis (Lambert et al. 2013). It follows that all the AD GWASs failed to detect the association, not because there is no association, but because the GWAS must be underpowered to detect it.

One explanation for this is that an association can be missed due to a recessive effect. A limitation of most GWASs is that they utilize a one-degree of freedom test optimal for detecting an additive disease model, but which performs poorly if the actual disease model is recessive (Lettre et al. 2007). We find that the size of this variant’s recessive effect (OR_AA_ = 1.73) is concealed when only considering its additive or per-allele effect size (OR_A_ = 1.15). Herein, we propose that this explanation also serves as a hypothesis to account for some of the current “missing heritability” for common diseases. For AMD only 15–65 % of total heritability is explained by the 19 loci detected so far (Fritsche et al. 2013). A number of hypotheses have sought to predict the nature of the undetected genetic variants that account for this considerable missing heritability. One hypothesis proposes that it is due to common variants with weak effect, also known as the infinitesimal model (Gibson 2011) and has a growing body of supporting evidence (Hunt et al. 2013). We propose that a subset of these common variants with weak effect are likely to be common variants with recessive effect (CVRE). We consider this distinction important as it gives further promise for detecting additional associated variants using currently employed sample sizes. Although a common recessive variant may be considered weak using an additive model, its recessive effect (OR_NN_) can be much stronger (Fig. 2) and therefore could be detected using an appropriately designed test. We predict that it will be very informative to analyse existing GWAS datasets to test specifically for recessive effects. We consider the CVRE hypothesis is consistent with the knowledge that there are many simple genetic diseases known to be recessive, and that recessive variants are now beginning to be found in complex diseases (Yang et al. 2012). Indeed other candidate gene studies of AMD have discovered associated variants with recessive effect (Jun et al. 2011), which were not detected by the AMD GWASs. The CVRE hypothesis is also consistent with the fact that a recessive variant is more likely to rise to a common allele frequency than a dominant or additive variant because it is under less selective pressure (Curtis 2013).

We present evidence that *CST3* is a shared genetic risk factor for both AMD and AD. It was anticipated that variants linked to AMD may contribute to other prevalent age-related diseases involving chronic, local inflammatory processes (Hageman 2012). It has also been documented that both AD plaques and AMD drusen involve amyloid-β peptides and the complex enzymatic systems necessary to generate them (Zhao et al. 2014). A well-known gene implicated in both diseases is *APOE*. Interestingly however this actually exhibits antagonistic pleiotropy, whereas the ε4 allele increases an individual’s AD risk it decreases AMD risk. Due to this and other unshared risk factors, we do not expect the shared association of *CST3* to be sufficient to cause comorbidity between AD and AMD. Indeed a recent study did not find a significant shared incidence between the two diseases (Keenan et al. 2014). However, this is not to say this is a research opportunity not worth exploring; understanding more about the functional mechanism of cystatin C and its associated cellular pathways may provide insights into both diseases, and identify further molecular targets for treatment and prevention. Furthermore, the recessive nature may be favourable with respect to therapeutics; a number of autosomal recessive diseases have already been successfully treated using replacement therapy. Replacing the dysfunctional or deficient gene with a functional copy has been achieved by administering the functional protein (Escobar 2013) and more recently by using gene therapy (Gaudet et al. 2013).

**Fig. 5:**
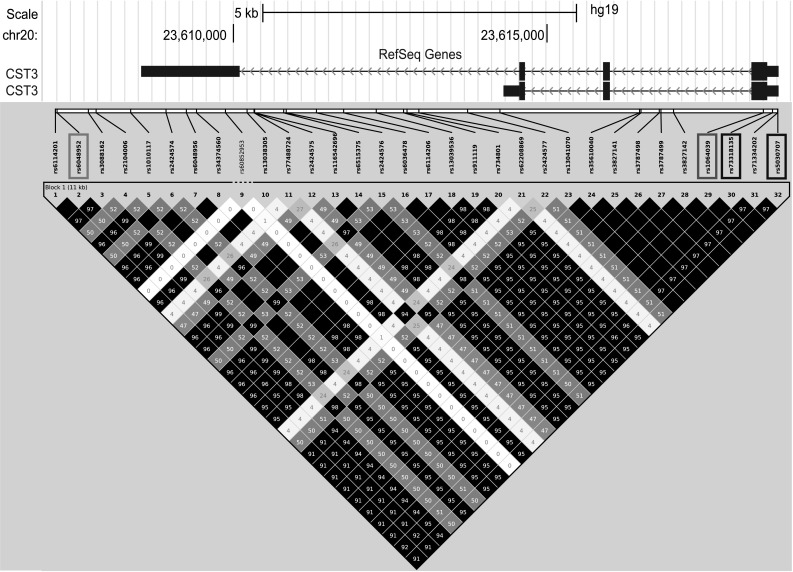
Pairwise linkage disequilibrium map of *CST3* SNPs (maf >0.05) from a Caucasian sample (*n* = 503, from Phase 3 of the 1000 Genomes Project). *Solid black squares* represent pairs of SNPs in high LD (*R* ^2^ > 0.9) as depicted by Haploview. Missense SNP highlighted in *red*, the two other SNPs in the PCR product highlighted in *blue*, and the SNP associated with plasma level of cystatin C highlighted in *green* (colour figure online)

Further support for the *CST3* nsSNP having a functional role comes from a recent GWAS that detected an association (*P* = 7.82 × 10^−16^) between an SNP 1.3 kb downstream of *CST3* (rs6048952) and plasma levels of cystatin C (Akerblom et al. 2014). We found the variant that corresponds to decreased plasma cystatin C is on the same haplotype as the AMD/AD risk allele rs1064039-A (pairwise LD: *R*^2^ = 0.92, *D*′ = 0.99) (Fig. 5). This observation presents a mechanistic link between genotype and disease phenotype and it also lends further support to the idea that cystatin C replacement therapy may be a fruitful therapeutic avenue. We maintain that the rs1064039 polymorphism is the driver of the reduced secretion because transfection of RPE cells with a construct encoding a different amino acid (serine) at that position leads to an intermediate level of secretion, between the wild type (alanine) and variant B (threonine) levels (Ratnayaka et al. 2007). Decreased secretion of cystatin C has also been observed in fibroblasts taken from AD donors homozygous for variant B when compared with fibroblasts from AD donors heterozygous or wild-type homozygous (Benussi et al. 2003).

In conclusion, we present evidence that strengthens the hypothesis that *CST3* is implicated in AMD pathogenesis. In particular, only individuals homozygous for the variant allele are at increased risk. Intriguingly the same recessive effect is observed at the same SNP with AD risk. This finding corresponds with previous evidence from both AD and AMD in vitro models. Observing a recessive effect implies that a single wild-type allele is able to compensate for the mutant allele. This may be due to cystatin C being a potent inhibitor of cysteine proteases (inhibitory constant *k*_*i*_ for cathepsin B is 0.25 nM) (Barrett et al. 1984). Therefore, gene expression from a single wild-type copy is expected to maintain proteolytic homeostasis, whereas absence of both wild-type copies is likely to lead to proteolytic dysregulation. It is interesting to note that proteolytic dysregulation has been implicated in the pathogenesis of both AMD and AD (Kaarniranta et al. 2011). Specifically inhibition of cathepsin B has been shown to play an important role in improving memory function and reducing levels of β-amyloid in transgenic AD mice (Hook et al. 2008). It is also interesting that another protease inhibitor, *TIMP3*, has recently been linked with susceptibility to AMD (Ardeljan et al. 2013; Fritsche et al. 2013). Further research will be required to fully elucidate the roles of protease inhibitors with respect to AMD pathogenesis.

## Electronic supplementary material

Supplementary material 1 (PDF 191 kb)
