# Supplementary material for: A missense variant in CST3 exerts a recessive effect on susceptibility to age-related macular degeneration resembling its association with Alzheimer’s disease
Source: Hum Genet. 2015 Apr 19;134(7):705–15. doi: 10.1007/s00439-015-1552-7 (PMC4460273; doi:10.1007/s00439-015-1552-7)
Supplement: Supplementary file 1 — Supplementary material 1 (PDF 191 kb) [file 439_2015_1552_MOESM1_ESM.pdf]

ELECTRONIC SUPPLEMENTARY MATERIAL

A missense variant in *CST3* exerts a recessive effect on susceptibility to age-related macular degeneration resembling its association with Alzheimer's disease

Joe M. Butler · Umar Sharif · Manir Ali · Martin McKibbin · Joseph P. Thompson · Richard Gale · Yit C. Yang · Chris Inglehearn · Luminita Paraoan

J. M. Butler and U. Sharif share first authorship

J. M. Butler · U. Sharif · L. Paraoan (⌂)
Department of Eye and Vision Science, Institute of Ageing and Chronic Disease, University of Liverpool, Liverpool, L69 3GA, United Kingdom
E-mail: lparaoan@liv.ac.uk
Tel.: +44 151 7064101
Fax: +44 151 706 5934

M. Ali · J. P. Thompson · C. Inglehearn
Ophthalmology and Neuroscience, University of Leeds, Leeds, LS9 7TF, United Kingdom

M. McKibbin
Ophthalmology Department, St James's University Hospital, Leeds, LS9 7TF, United Kingdom

R. Gale
Ophthalmology Department, The York Hospital, York, YO31 8HE, United Kingdom

Y. C. Yang
Ophthalmology, The Royal Wolverhampton NHS Trust, Wolverhampton, WV10 0QP, United Kingdom.

Journal: Human Genetics

a

Study or Subgroup	AA Events	AA Total	GG Events	GG Total	Weight	Odds Ratio M-H, Fixed, 95% CI	Year
Finckh et al. (USA) (2000)	19	22	257	418	8.3%	3.97 [1.16, 13.62]	2000
Crawford et al. (2000)	16	23	183	263	21.3%	1.00 [0.40, 2.52]	2000
Finckh et al. (2000)	10	13	65	164	5.2%	5.08 [1.35, 19.15]	2000
Roks et al. (2001)	6	12	60	133	11.8%	1.22 [0.37, 3.97]	2001
Monastero et al. (2005)	8	15	120	245	15.3%	1.19 [0.42, 3.38]	2005
Beyer et al. (2005)	6	11	109	215	11.5%	1.17 [0.35, 3.94]	2005
Cathcart et al. (2005)	17	23	106	204	13.3%	2.62 [0.99, 6.91]	2005
Nacmias et al. (2006)	6	11	173	299	13.3%	0.87 [0.26, 2.93]	2006
**Total (95% CI)**		**130**		**1941**	**100.0%**	**1.73 [1.18, 2.54]**	
Total events	88		1073				

Heterogeneity: Chi² = 8.78, df = 7 (P = 0.27); I² = 20%
Test for overall effect: Z = 2.82 (P = 0.005)

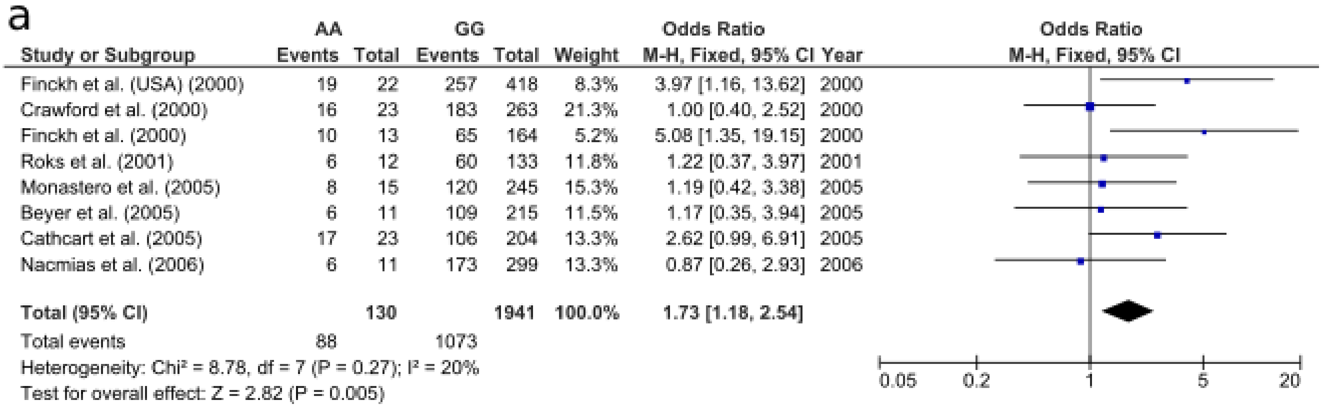

b

Study or Subgroup	AG Events	AG Total	GG Events	GG Total	Weight	Odds Ratio M-H, Fixed, 95% CI	Year
Finckh et al. (2000)	131	207	257	418	21.2%	1.08 [0.77, 1.52]	2000
Finckh et al. (USA) (2000)	35	83	65	164	8.6%	1.11 [0.65, 1.90]	2000
Crawford et al. (2000)	110	157	183	263	13.9%	1.02 [0.66, 1.57]	2000
Roks et al. (2001)	30	69	60	133	7.9%	0.94 [0.52, 1.68]	2001
Monastero et al. (2005)	63	123	120	245	13.3%	1.09 [0.71, 1.69]	2005
Cathcart et al. (2005)	56	93	106	204	9.0%	1.40 [0.85, 2.30]	2005
Beyer et al. (2005)	48	92	109	215	10.6%	1.06 [0.65, 1.73]	2005
Nacmias et al. (2006)	64	119	173	299	15.5%	0.85 [0.55, 1.30]	2006
**Total (95% CI)**		**943**		**1941**	**100.0%**	**1.06 [0.90, 1.24]**	
Total events	537		1073				

Heterogeneity: Chi² = 2.51, df = 7 (P = 0.93); I² = 0%
Test for overall effect: Z = 0.67 (P = 0.50)

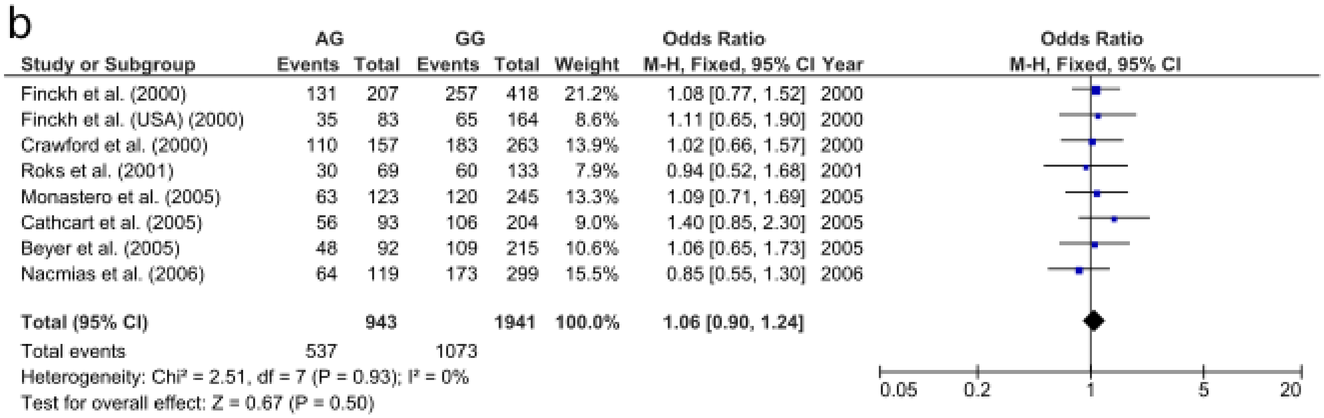

Fig. S1 Forest plots for the meta-analysis of *CST3* rs1064039 with respect to Alzheimer's disease in the Caucasian population using a fixed effects model. Area of the squares represents the weight of the study and horizontal bars represent 95% confidence interval of the OR. Applied to (**a**) "AA" genotype versus genotypes [data taken directly from Hua et al. (2012)] and (**b**) "AG" genotype versus "GG" genotype [data inferred from Hua et al. (2012)]

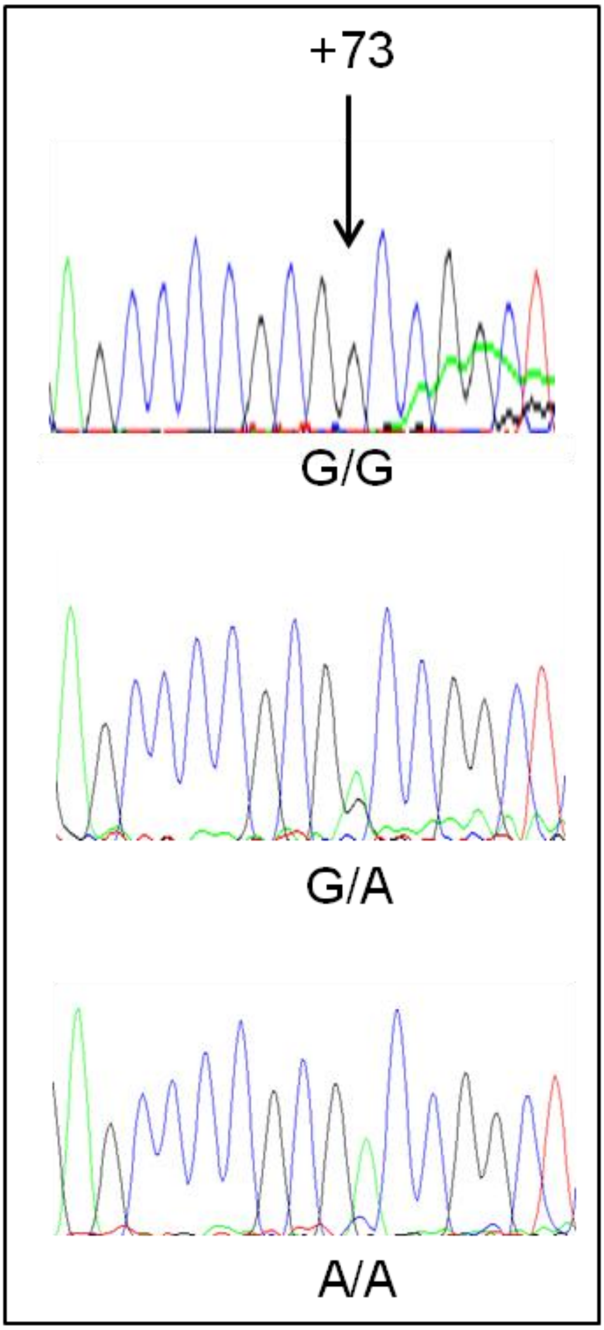

Fig. S2 Representative chromatograms of the three genotypes at rs1064039. Colour of peaks represents the different nucleotides (green=A, black=G, blue=C, red=T). The PCR products were generated using primers CST3RIIF and CST3LIIR and sequenced using CST3BIIR
